# Can integration reduce inequity in healthcare utilization? Evidence and hurdles in China

**DOI:** 10.1186/s12913-019-4480-8

**Published:** 2019-09-10

**Authors:** Miaomiao Zhao, Baohua Liu, Linghan Shan, Cui Li, Qunhong Wu, Yanhua Hao, Zhuo Chen, Lan Lan, Zheng Kang, Libo Liang, Ning Ning, Mingli Jiao

**Affiliations:** 1 Department of Social Medicine, School of Health Management, Harbin Medical University, 157 Baojian Road, Nangang District, Harbin, 150086 Heilongjiang China; 2 Department of Health Management, School of Public Health, Nantong University, 9 Seyuan Road, Chongchuan District, Nantong, 226019 Jiangsu China; 3 Department of Health Policy and Management College of Public Health, University of Georgia, Athens, GA 30602 USA; 4 School of Economics, Faculty of Humanities and Social Sciences, University of Nottingham Ningbo, 199 Taikang East Road, Ningbo, Zhejiang, 315100 China

**Keywords:** Medical insurance integration, Inequity, Inequality, Inpatient service utilization

## Abstract

**Background:**

Integration of medical insurance schemes has been prioritized as one of the key strategies to address inequity in China’s health system. The first pilot attempt to integrate started in 2003 and later expanded nationwide. This study aims to assess its intended impact on inequity in inpatient service utilization and identify the main determinants contributing to its ineffectiveness.

**Methods:**

A total of 49,365 respondents in the pilot integrated area and 77,165 respondents in the non-integration area were extracted from the Fifth National Health Services Survey. A comparative analysis was conducted between two types of areas. We calculate a concentration index (CI) and horizontal inequity index (HI) in inpatient service utilization and decompose the two indices.

**Results:**

Insurance integration played a positive role in reducing inequality in inpatient service utilization to some extent. A 13.23% lower in HI, a decrease in unmet inpatient care and financial barriers to inpatient care in the pilot integrated area compared with the non-integration area; decomposition analysis showed that the Urban-Rural Residents Basic Medical Insurance, a type of integrated insurance, contributed 37.49% to reducing inequality in inpatient service utilization. However, it still could not offset the strong negative effect of income and other insurance schemes that have increased inequality.

**Conclusions:**

The earlier pilot attempt for integrating medical insurance was not enough to counteract the influence of factors which increased the inequality in inpatient service utilization. Further efforts to address the inequality should focus on widening access to financing, upgrading the risk pool, reducing gaps within and between insurance schemes, and providing broader chronic disease benefit packages. Social policies that target the needs of the poor with coordinated efforts from various levels and agencies of the government are urgently needed.

**Electronic supplementary material:**

The online version of this article (10.1186/s12913-019-4480-8) contains supplementary material, which is available to authorized users.

## Background

Equity in healthcare is one of the most important priorities in any healthcare system [1]. The achievement of equal access to healthcare is regarded as a key element of health system performance and universal health coverage (UHC) [2, 3]. Equity in healthcare utilization is affected not only by an individual’s socioeconomic status [4], but also by fragmentation in healthcare system [5]. Health insurance is one of the most important factors to improve equity in healthcare since it provides a safeguard against risks and barriers to healthcare especially for those who are suffering financial difficulty [6, 7]. In many cases, developing countries are striving to achieve UHC quickly by combining multiple health insurance schemes covering different population groups into fewer or a unified insurance scheme [8]. Meanwhile, differences in government subsidies of the premium and benefit packages offered by the fragmented insurance scheme unexpectedly contribute to inequity in healthcare utilization across groups with different levels of wealth [9, 10]. A study using data from nine developing countries shows the gap of healthcare utilization between the richest and the poorest ranged from 1.7 times to a surprising 12 times [11].

China achieved the goal of universal coverage of medical insurance swiftly by establishing multiple medical schemes. Since the introduction of social medical insurance in 1990s, insurance coverage gradually expanded. By 2011, about 95% of the Chinese population was covered by a Social Basic Medical Insurance (SBMI) programme including the Urban Employee Basic Medical Insurance (UEBMI), the New Rural Cooperative Medical Scheme (NRCMS), and the Urban Resident Basic Medical Insurance (URBMI) [12]. This achievement is considered to be the first step toward UHC [13]. However, the SBMI system in China was highly segmented. The three schemes were separately administered and operated locally based on different eligibility requirements (employment status, urban and rural household registration) [14]. The UEBMI covered urban employees with the funds contributed from the employers and employees going into a collectively pooled account at the municipal level and an individual medical savings account. The NRCMS was a voluntary medical insurance program for rural residents, which was jointly funded by government subsidies and individual premiums at county-level. The URBMI was for urban residents who were not covered by the UEBMI or NRCMS, and the funds were pooled at the municipal-level with contributions from government subsidies and premiums. Therefore, more than 3000 funds operated independently in the three SBMI schemes [10]. The benefits package of services and medicines provided as well as reimbursement policy varied significantly among different insurance types, resulting in a rapid increase of inequity in healthcare utilization. Previous studies revealed a gap of 2.33 times in actual use of inpatient services between richest and poorest [15].

In order to address the inequity problem caused by the fragmented medical insurance system, the Chinese government selected several areas to launch pilot insurance integration reform since 2003. Two kinds of models emerged in the pilot areas. The one which merged the UEBMI, URBMI, and NRCMS into one uniform scheme called Uniform Social Basic Medical Insurance (USBMI). This model was adopted by several high-income cities such as Zhongshan and Dongguan [16, 17]. The other type of pilot only merged the URBMI and NRCMS and is called Urban-Rural Residents Basic Medical Insurance (URRBMI). URRBMI was adopted by most pilots because the financing source and level of contribution of the URBMI and NRCMS were roughly similar. Aspects including unifying enrollees, premiums, pooling level of fund, benefits packages, reimbursements arrangement, and fund management system were involved in reform [18]. With the health reform deepening and fragmentation more recognizable nationwide, Chinese government officially endorsed a nationwide policy for medical insurance integration in 2016 [19]. Currently, the national integration reform is still in its initial stage, facing many challenges because of the absence of national guideline. Thus it is timely and fills a critical need to conduct studies about the integration pilots.

Current studies on integration reform mainly focus on a theoretical policy analysis [14, 20], and the observational summary of the pilots’ experience [21–23]. Some empirical research studied the willingness and satisfaction among enrollees towards integration [18, 24]. Equity in healthcare utilization is advocated as the core goal of integrated reform. However, there is no quantitative study so far that used national representative data to evaluate to what degree this objective has been achieved. Using inequity in inpatient service utilization as the main variable of interest, this study seeks to answer the following questions: how is the inequity in inpatient service utilization in the integrated area comparing with that in the non-integrated area? What is the impact of the insurance integration on inequity? What’s the hurdle to implementing insurance integration and how to improve it? Our findings will provide evidential support for future policy development on insurance integration in China and offer lessons to countries that are facing similar challenges.

## Method

### Data source

The data was drawn from the Fifth National Health Services Survey (NHSS) in 2013. A multi-stage stratified cluster random sampling method was used and all the responses were self-reported. The NHSS covered 31 provinces with 156 sample areas (including nearly 300,000 respondents from 93,600 households).

In NHSS, there were 10 provinces whose sample areas had both integration reform pilots and non-integrated areas; 22 pilot areas in these 10 provinces were grouped as the integration group (pilot integrated area) and the remaining 42 non-integrated areas were grouped as the reference group (non-integrated area). In addition, the integration group also include 3 provinces that underwent a total integration reform (therefore has no reference group), we chose 3 other provinces whose sample areas were all without integration reform but with similar social economic levels (per capita GDP) as their reference group. The features and differences of the medical insurance schemes in pilot integration area and non-integrated area are shown in Table 1. Finally, 49,365 respondents from the integrated area and 77,165 respondents from the non-integrated area were sampled.

**Table 1:** Comparison of the medical insurance schemes and its features and differences in integration area and non-integration area

	integration pilot area		Non-integration area
	Model 1	Model 2	
Specific arrangement	Merged the three existing insurance schemes (UEBMI, URBMI and NRCMS) into a new scheme--USBMI	Only merged the URBMI and NRCMS into a new scheme—URRBMI, UEBMI was kept.	The three existing insurance schemes still run separately.
Population coverage	USBMI covered all population	URRBMI covered urban-rural residents except for urban employees.	UEBMI covered urban employees, URBMI covered urban residents, NRCMS covered rural residents.
Pooling level of fund	USBMI was pooled at municipal level.	URRBMI were pooled at municipal level.	UEBMI and URBMI were pooled at municipal level, while NRCMS was pooled at county level.
Contribution of premium	All urban employees kept the previous percentage of wage for premium contribution which was shared by employees and employers; the remaining urban and rural residents paid the flat rate, which was also shared by local government.	In URRBMI, urban-rural residents paid uniform flat rate and was shared by individual and government, which was adjusted yearly; the premium level of URRBMI were much higher than un-integrated insurance schemes.	In UEBMI, employees paid percentage of wage for premium contribution which were shared by employees and employers; In URBMI and NRCMS, residents paid the flat rate, which was also shared by local government; the premium level of URBMI was higher than NRCMS
Fund management	All the funds were eventually pooled together and were uniformly managed.	The fund of URRBMI were uniformly managed but were separated from UEBMI.	The fund of three schemes were separately managed
Benefit package	The benefit package was expanded compared to the previous schemes and was unified for all enrollees.	In URRBMI, the benefit package was expanded compared to the previous URBMI and NRCMS and was unified for urban and rural residents.	UEBMI> URBMI>NRCMS
Reimbursement rate	The reimbursement rare was higher than previous schemes and was unified for all enrollees.	In URRBMI, reimbursement rare was higher than previous URBMI and NRCMS and was unified for urban and rural residents.	UEBMI> URBMI>NRCMS

### Variables definition

**Inpatient service utilization** referred to the use of inpatient service in the previous year, which is based on the question “have you been hospitalized in the past year?”

**Need factors** included sex, age, self-assessed health, chronic conditions and functional limitation etc.

**Non-need factors** were other socio-economic variables which influenced use of healthcare except need factors, which included socioeconomic status, education, occupation, household income, region, and medical insurance etc.

### Analytic approach

In this study, we used a concentration index (CI) to measure the degree of income-related inequality which was derived from the concentration curve that plots the cumulative health care utilization against the cumulative distribution of population ranked by socioeconomic status such as income. CI was further decomposed to assess the contribution of different factors (need factors and non-need factors) in explaining inequality in inpatient service utilization. The horizontal inequity (HI) index indicated the income-related inequity in health care utilization after standardizing for differences in health need, such as sex, age and health conditions. HI was calculated based on the CI decomposition results. These methods were proposed by Wagstaff [25, 26] and extensively used by many researchers [27–34]. The calculation steps were as follows:

- **Step 1 Standardization of inpatient service utilization**

Three groups of utilizations including actual inpatient service utilization, need-predicted inpatient service utilization, and need-standardized inpatient service utilization were calculated. Actual inpatient service utilization was collected in NHSS. Need-predicted inpatient service utilization was calculated through statistical modeling, aiming to capture variation in utilization predicted only by needs for inpatient service. Need-standardized inpatient service utilization was used to measure the gap between actual inpatient service utilization and need-predicted inpatient service utilization [32]. An indirect standardization with probit regression model was used to calculate the distribution of need-standardized inpatient service utilization as it was binary [25].

- **Step 2 Estimate of CI and its decomposition**

The CI index is calculated through equation following [25]:

$$ \mathrm{CI}=\frac{2}{\mu}\mathit{\operatorname{cov}}\left(h,r\right) $$

Where *h* is need-standardized inpatient service utilization, *μ* is the mean of need-standardized inpatient service utilization, *r* is the fractional rank of the individual by income.

The CI is decomposed into contributions of need factors and non-need factors based on probit regression model [31].

$$ {y}_i=\alpha +\sum \limits_j{\beta}_j^m{x}_{ji}+\sum \limits_k{\gamma}_k^n{z}_{ki}+{\varepsilon}_i $$

Where *y*_*i*_ is the probability of inpatient service utilization; *x*_*ji*_ are the need factors; *z*_*ki*_ are the non-need factors; ${\beta}_j^m$ and ${\gamma}_k^n$ are marginal effects of each variable; *ε*_*i*_ is the error term.

- **Step 3 Calculation of the HI**

HI is computed by subtracting the contribution of need factors from the CI, reflecting the degree to which inpatient care service is distributed by income after standardizing for differences in health need [26].

$$ \mathrm{HI}={CI}_m-{CI}_n $$

Where *CI*_*m*_ refers to the CI of actual inpatient service utilization, *CI*_*n*_ refers to the CI of the need-expected inpatient service utilization.

All analyses were performed in Stata 12.1.

## Results

### Description of the survey population

Both in the pilot integrated area and non-integrated area, the survey population was predominantly 45 years old and above, married and employed. In the pilot integrated area, 27.48% of respondents were covered by UEBMI, 5.47% by USBMI, 57.63% by URRBMI, and 5.86% covered by mixed-insurance (enrolled in both social medical insurance and commercial medical insurance). While in the non-integrated area, 23.71% covered by UEBMI, 10.43% by URBMI, 56.63% by NRCMS, and 5.88% covered by mixed-insurance (see Additional file 1).

### Distribution of inpatient service utilization across household income quintiles

**Fig. 1:**
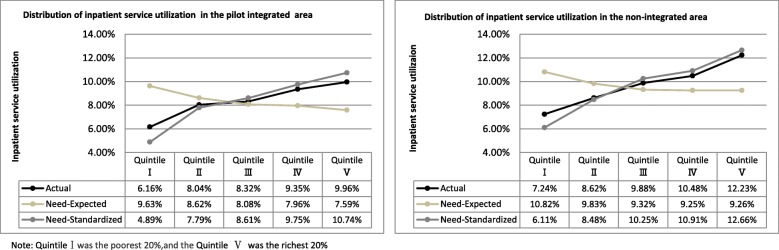
Distribution of inpatient service utilization across household income quintiles

Figure 1 showed the inpatient service utilization by household income quintiles. The actual inpatient service utilization reported by the richest group was 1.6 times of the poorest in the pilot integrated area, and 1.7 times in the non-integrated area. It demonstrated a narrower gap of actual inpatient service utilization between the rich and the poor in the pilot integrated area.

**Fig. 2:**
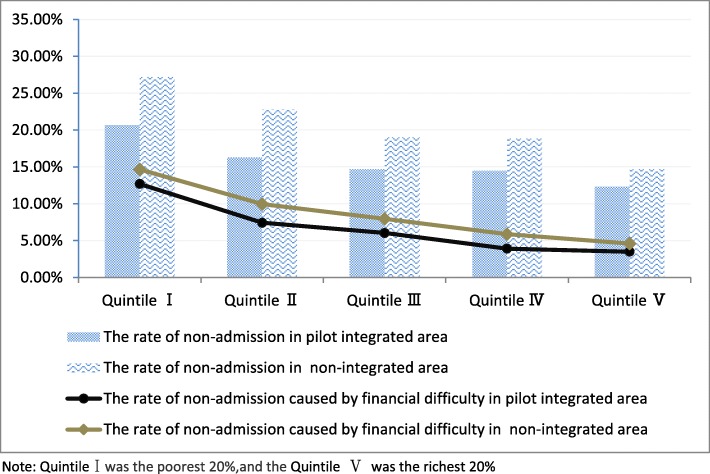
Non-admission rates across household income quintiles

The quintile distribution also shows the difference between the actual and need-expected inpatient service utilization. The actual inpatient service utilization by the richest and the second richest group was about 1.31 times and 1.17 times of their need-expected inpatient service utilization in the pilot integrated area, the figure in non-integrated area was 1.32 times and 1.13 times. While the actual inpatient service utilization by the poorest and second poorest group accounted for 63.97 and 93.27% of their need-expected inpatient service utilization in the pilot integrated area respectively, accounting for 66.91 and 87.69% in the non-integrated area. It demonstrated that the overall level of overuse inpatient service among the rich in the pilot integrated area was nearly the same as that in non-integrated area, and the underuse was much lower in second poorest group in the pilot integrated area than non-integrated area although the underuse among the poorest group was slightly higher.

### Distribution of the non-admission across household income quintiles

Both in the pilot integrated area and non-integrated area, the no-admission rate (defined as the percentage of patients needing hospitalization but unable to obtain it for various reasons) and the no-admission rate caused by financial difficulty all decreased with rising income quintiles, indicating that the poor group were more likely to forgo needed hospitalization. Nevertheless, the overall no-admission rate and the no-admission rate caused by financial difficulty in the pilot integrated area were all lower than in the non-integrated area among each quintile (average difference was − 4.50 and − 1.10% respectively). More substantial reductions were observed in the poorest and second poorest group (the differences for no-admission rate were − 6.52% and − 6.47%, for the no-admission rate caused by financial difficulty were − 1.97 and − 2.51%). Which shown that the pilot integrated area enjoy much reduced no-admission rate and financial barrier (see Fig. 2).

### Inequality and inequity in inpatient service utilization

Table 2 shows the results of the CI and HI indexes. The actual distribution of inpatient service utilization was pro-rich in the two areas, while the CI was 15.67% lower in the pilot integrated area (CI_M_ = 0.0877) than the non-integrated area (CI_M_ = 0.1040). After need was taken into account, the HI showed even more pro-rich inequity in two areas, however the inequity degree in the pilot integrated area (HI = 0.0984) was 13.23% lower than non-integrated area (HI = 0.1134). This indicated the inequity degree of inpatient service utilization was reduced in the pilot integrated area.

**Table 2:** CI and HI index of inpatient utilization

	CI_M_ (Actual)	CI_N_ (Need-Expected)	HI (Need-Standardized)
Pilot integrated area	0.0877^*^	−0.0107^*^	0.0984^*^
Non-integrated area	0.1040^*^	−0.0094^*^	0.1134^*^

^*^p < 0.05

### Decomposition of inequality in inpatient service utilization

Table 3 shows the results of the decomposition analysis, including each determinant’s marginal effect and CI_k_ and contribution to CI.

**Table 3:** Decomposition of inequality in inpatient service utilization

	Pilot integrated area			Non-integrated area		
	Marginal effect (β_k_)	CI_k_	Contribution to CI (%)	Marginal effect (β_k_)	CI_k_	Contribution to CI (%)
Sex and Age						
Male						
15–24(reference)						
25–34	− 0.033^**^	0.063	−2.00%	−0.026^**^	− 0.010	0.16%
35–44	− 0.026^**^	0.073	−2.20%	−0.009	0.076	−0.56%
45–54	− 0.017^*^	0.043	−0.93%	−.0003	0.053	−0.02%
55–64	−0.006	−0.071	0.62%	0.028^**^	−0.065	−1.78%
65−	0.019^*^	−0.106	−2.49%	0.058^**^	−0.065	−3.43%
Female						
15–24	0.109^**^	0.008	0.64%	0.160^**^	−0.010	− 0.78%
25–34	0.082^**^	0.101	8.57%	0.119^**^	0.028	2.28%
35–44	−0.011	0.078	−1.05%	0 .012	0.096	1.07%
45–54	− 0.013	0.022	−0.38%	0.009	0.042	0.41%
55–64	−0.008	−0.046	0.51%	0.026^**^	−0.060	−1.61%
65−	0.015	−0.124	−2.35%	0.053^**^	−0.064	−3.08%
Chronic disease						
Yes	0.066^**^	0.021	4.67%	0.095^**^	0.035	7.76%
No (reference)						
Limitation of daily activities						
Yes	0.060^**^	−0.126	−4.95%	0.041^**^	− 0.124	−2.64%
No (reference)						
Self−assessment health						
Very poor	0.155^**^	−0.115	−0.65%	0.113^**^	−0.038	−0.15%
Poor	0.099^**^	−0.116	−2.35%	0.124^**^	−0.079	−1.59%
Medium	0.078^**^	−0.067	−6.55%	0.074^**^	−0.064	−5.31%
Good	0.027^**^	−0.010	−1.35%	0.023^**^	0.003	0.25%
Very good (reference)						
Education						
Illiterate (reference)						
Primary school	0.004	−0.083	−2.74%	0.006	−0.086	−2.77%
Secondary school	−0.06	0.138	−2.17%	−0.002	0.179	−0.69%
College and above	−0.015^**^	0.389	−10.81%	−0.008	0.442	−4.02%
Occupation status						
Unemployment (reference)						
Student	−0.055^**^	0.059	−2.09%	−0.066^**^	0.150	−4.13%
Peasant	−0.004	−0.296	4.21%	0.004	−0.201	−3.13%
Worker	−0.026^**^	0.100	−3.44%	−0.015^**^	0.189	− 1.70%
Business	−0.031^**^	0.181	−9.75%	−0.023^**^	0.200	−4.54%
Manager	−0.027^**^	0.293	−22.35%	−0.016^**^	0.351	−9.71%
Other	−0.017^**^	0.052	−1.16%	−0.017^**^	0.062	−0.93%
Marital status						
Other (reference)						
Married	0.021^**^	0.015	3.32%	0.022^**^	0.014	2.39%
Household income						
Quintile I (reference)						
Quintile II	0.033^**^	−0.400	−36.32%	0.028^**^	−0.400	−22.00%
Quintile III	0.046^**^	0.000	0.04%	0.048^**^	0.000	0.02%
Quintile IV	0.059^**^	0.401	65.21%	0.059^**^	0.400	47.01%
Quintile V	0.075^**^	0.801	162.78%	0.081^**^	0.800	128.84%
Medical insurance						
UEBMI	0.040^**^	0.248	37.50%	0.030^**^	0.355	25.39%
URBMI				0.017^*^	0.043	0.74%
NRCMS				0.014^*^	−0.184	−14.17%
USBMI	0.029^**^	0.277	6.07%			
URRBMI	0.027^**^	−0.175	−37.49%			
Mixed−insurance	0.046^**^	0.269	9.90%	0.025^**^	0.245	3.54%
Uninsured and other (reference)						
Distance to the nearest health facilities						
< 1 km(reference)						
1 − 4 km	0.008^**^	−0.061	−2.56%	0.008^**^	−0.101	−2.77%
≥ 5 km	0.019^**^	−0.122	−0.82%	0.046^**^	−0.228	−2.57%
Time to the nearest health facilities						
< 15 min (reference)						
15 − 29 min	−0.003	−0.063	0.47%	−0.004	−0.067	0.54%
≥ 30 min	−0.005	−0.265	0.90%	−0.004	−0.288	0.77%
Preferred health facilities						
Primary facilities (reference)						
Non − primary facilities	0.004	0.223	3.00%	0.006^*^	0.321	3.63%
Residence						
Urban (reference)						
Rural	0.005^*^	−0.196	−6.52%	0.013^**^	−0.192	−12.69%
Region						
Eastern	−0.029^**^	0.059	−13.05%	−0.038^**^	0.087	−10.12%
Middle	−0.014^**^	−0.135	3.13%	−0.013^**^	−0.002	0.09%
Western (reference)						

Quintile I was the poorest 20%,and the Quintile V was the richest 20%

Note:^*^p < 0.05; ^**^p < 0.01

The marginal effect denotes the association between the determinants and the inpatient service utilization. A positive marginal effect means that that factor promoted utilization, and vice versa. Both in the pilot integrated area and non-integrated area, medical insurance (regardless of type) can significantly increase the inpatient service utilization compared to the uninsured group. The UEBMI and mixed-insurance are two of most important factors that significantly increased the inpatient service utilization in the two areas. In addition, the role in increasing inpatient service utilization played by USBMI and URRBMI in the pilot integrated area was higher than URBMI and NRCMS in the non-integrated area.

The CI_k_ was employed to describe how each determinant was distributed (range from − 1~ + 1) over the factor of wealth. With regard to the medical insurance type, the URRBMI (CI_k_ = − 0.175) and NRCMS (CI_k_ = − 0.184) were more concentrated among the poor, while the USBMI, UEBMI, URBMI, and mixed-insurance were more concentrated among the rich.

The contribution to CI describes each determinant’s role in inequality. A positive value implies the determinant increased inequality, and vice versa. In both areas, household income accounted for most of the inequalities (162.78 and 128.84% for the highest quintile in the pilot integrated area and non-integrated area respectively, it is also true for the second highest quintile groups). While in the pilot integrated area, URRBMI has a pro-poor contribution (− 37.49%), meaning it played a positive role in reducing inequity by enhancing more inpatient service utilization among poor population. But the other insurance including USBMI and mixed-insurance all contributed from 6.08 to 37.51%, especially UEBMI contribute 37.51% to the pro-rich inequity. Moreover, chronic disease made a pro-rich contribution in the pilot integrated area (4.67%) and non-integrated area (7.76%), while remaining need factors made pro-poor contributions. In addition, rural area (− 6.52 and − 12.69%) and eastern region (− 13.05 and − 10.12%) had pro-poor contributions that achieving better performance in reducing inequality.

## Discussion

Medical insurance integration reform is one of the key strategies addressing inequity issues caused by a fragmented health system in China. This study compares the pilot integrated area and non-integrated area by employing nationally representative data, and provides powerful evidence of the effectiveness of integration reform in achieving the primary goal of reducing inequity. It also provides a comprehensive view of the combined role of medical insurance with other demographic and socioeconomic factors through a decomposition analysis.

The study reveals a mixed picture in terms of the distribution of inpatient service utilization and how insurance integration influences the degree of inequality. Through comparisons, we found the gap of inpatient service utilization between the rich and the poor was narrower in the pilot integrated area than in the non-integrated area (1.61 times vs 1.69 times). Whilst the non-admission rate and the non-admission rate caused by financial difficulty were all lower in the pilot integrated area than in the non-integrated area across each household income quintile (average differences were − 4.50 and − 1.10% respectively), the reduced gaps were even larger in the poorest and second poorest group (the differences for no-admission rate were − 6.52 and − 6.47%, for the no-admission rate caused by financial difficulty were − 1.97 and − 2.51%), which indicates that the pilot integrated area enjoyed much reduced no-admission and financial barriers. In addition, the pro-rich equity of inpatient service utilization in the pilot integrated area was 13.23% lower than in the non-integrated area. Further, decomposition results show that the URRBMI made the greatest contribution in reducing the inequality (−37.49%) in the pilot integrated area although other insurance schemes increased the inequality. These findings to some extent revealed the positive impact of integration reform on reducing inequity in inpatient service utilization.

Integration reform could reduce inpatient service utilization inequity for several reasons. First, it connects different targeted populations regardless of their identity, occupation, and district [14]. That was essential to narrow the insurance benefit gaps and reduce the inequity in healthcare use. Second, for integrated insurance, the level of the benefit package and reimbursement rate was standardized and all increased, which was crucial to provide equitable financial protection to all beneficiaries. Third, risk pooling was increased after integration; for instance, the pooling level of URRBMI which merged the URBMI and NRCMS (previously mainly run at county level) was upgraded to the municipal level. The elevated funding pool will definitely increase the ability of integrated insurance funds to protect against risks [35], which may lead to more equitable access to inpatient health services.

Despite these encouraging results, inpatient service utilization was still pro-rich, and the poorest group still had some inpatient health service needs that were not met. Furthermore, in the decomposition analysis, we found the role played by URRBMI in reducing inequality could not counteract the role played by income or the presence of other insurance types (especially UEBMI) in increasing inequality. These results could be explained in two ways. One is in the imperfect design of the integration reform itself. The financing of integrated URRBMI was similar to that of NRCMS and URBMI (a flat rate contributed by individual and government), and despite increases in premiums, the financing ability of URRBMI has not improved markedly. In fact, the financing level of UEBMI was still nearly 10 times higher than URRBMI [36, 37]. The disparity in financing eventually led to the disparity in reimbursement levels, we found the average actual reimbursement rate of UEBMI was 66.8% while the URRMBI was 49.8% in 2013 (see Additional file 2). Thus, URRBMI did equalize the financing and reimbursement level between urban and rural residents and indeed provided more reimbursement for the poor than the rich (actual reimbursement rate range from 57.6% for the poorest to 44.6% for the richest), but the gap between it and UEBMI persists. The other reason why the URRBMI could not eliminate inequality was due to its stepwise implementation process. Many pilots are still in the first step of integrating the administration system, insurance agencies and funds, in order to reduce the resistance to reform and to reach the policy aim more easily [10]. In some piloted areas, the insurance fund still operates and is managed independently instead of being integrated into a uniform risk pool; this hinders the attainment of equity [20, 38]. Furthermore, most pilots provide two or three levels of premium – a higher level of premium means higher reimbursement and more subsidies [39]. But problems emerge. On the one hand, the arrangement of differential compensation based on the capacity to pay the premiums might transfer existing inequity from different insurance schemes into inequity within the integrated scheme. On the other hand, in a voluntary enrollment context, adverse selection may occur which goes against the financial sustainability of the insurance scheme [40].

Obstacles also emerged due to the negative role played by UEBMI in increasing inequity. Although the premium level of UEBMI was higher on average, the disparity of financing levels was outstanding among different areas. It could partly explain the contradictory results in the pilot integrated area: despite much reduced inequity level a pro-rich tendency persisted. The UEBMI contributed 37.50% to increase the inpatient service utilization inequality in the pilot integrated area. To overcome this issue more thoroughly, a better choice for China would be to merge all existing medical insurance into one scheme—USBMI, that was regarded as the ultimate goal of integration [39]. USBMI could achieve the equity goal through covering all enrollees by a single medical insurance scheme, since the uniformed and expanded fund pooling could increase the anti-risk ability of insurance funds [35]. Our results show the effect of USBMI on increasing inequality (contribution = 6.07%) was significantly lower than the existing UEBMI scheme no matter whether we examined the pilot integrated area (contribution = 37.50%) or non-integrated area (contribution = 25.39%). Nevertheless, due to the requirement for much higher level of premiums, greater government subsidies, a better fund management capacity and information system, USBMI currently is only piloted in a few highly developed areas, and not expanded nationally. Despite rapid socio-economic development in China, the income gap between the rich and the poor is widening; the income Gini coefficient is consistently higher than 0.47 since 2003 [41]. Although the benefits package in integrated insurance was provided equally to all enrollees, medical cost affordability remains different due to different household income [42]. This actually led to a gap in the ability to pay for health services between the rich and the poor and eventually caused inequity in access to healthcare. In this study, household income itself contributed 191.71 and 153.87% to the total inequalities in the pilot integrated area and the non-integrated area. Meanwhile, more than 12% of the poorest respondents had forgone hospitalization due to financial difficulty while the figure of the richest respondents was less than 5%. Due to insufficient assistance for the low income group by the existing social insurance and welfare policies, healthcare utilization equity cannot be achieved by integration reform itself, but requires a concerted multi-sectoral action [43].

Owing to the combined effort of medical insurance and other socioeconomic factors, urban-rural and regional disparities in healthcare have been reduced to some extent. This study shows that residing in the eastern region of the country reduced the inequality by 13.05% in the pilot integrated area, which was higher than it in the non-integrated area (10.12%). One possible explanation is that most integrated pilots were concentrated in the eastern region with the combined effect of developing both the economy and integration which have reinforced each other, leading to a reduction in inequality in this region. But for residents of rural areas, their inequality was reduced by 6.52% in the pilot integrated area which was much smaller than in the non-integrated area (12.69%). This might be partly explained by the fact that the URRBMI allowed rural residents to approach expensive urban health services, which thus reduced inpatient services use in the pilot integrated area.

An interesting finding of our study is that, among all the need factors, only the presence of chronic diseases drives inequality in inpatient service utilization. Such a phenomenon can be explained by the fact that chronic diseases are more concentrated among the rich; therefore, they used more inpatient services than the poor. The number of chronic disease patients in China is around 300 million [44] and chronic diseases account for approximate 90% of total deaths [45], posing a profound challenge for China’s healthcare system. The current medical insurance system, whether integrated or not, generally implements inpatient treatment-oriented benefits packages, neglecting prevention and outpatient services. Such arrangements easily lead to delayed treatment among the poor. Although many integrated pilots provide outpatient chronic disease packages, the number of conditions covered is usually limited 8~15 [46]. A shift to primary health care and a broader benefit package covering chronic disease management and treatment should be incorporated into future integration reform.

There were three limitations to the study. First, this was a cross-sectional study so the causal relationship between integration reform and measured factors could not be established. Second, the changes and effectiveness caused by insurance integration reform cannot be fully measured and demonstrated because this study only investigates the earlier stage of the pilot areas while the policy effect of integration reform usually takes longer to be fully revealed. Third, although the selected pilot integrated area and non-integrated area are from the same province or other provinces with similar socioeconomic levels to control the influence of socioeconomic factor, there still might be other socioeconomic factors that confound our results. However, this study provides rare and valuable policy evidence to evaluate the ongoing large scale medical insurance integration reform in China, through comparative analyses and quantitatively measuring the reduction in inequities resulting from integration reform and other influencing factors. It suggests options for more targeted policy interventions to address persistent problems.

## Conclusion

Integration reform played a positive role in reducing inequality in inpatient service utilization. However, inequality still exists, particularly among the poorer population. Improvements can be made. We offer some policy implications for China’s integration reform. First, against the backdrop of huge social, economic, geographical disparity in China, the implementation of nationwide uniform medical insurance scheme like USBMI is not likely to be completed within the short term. To reduce resistance to integration reform, URRBMI might be a more feasible and appropriate policy choice for China’s next stage of insurance reform. Second, to reduce the gap between URRBMI and UEBMI, governments at different levels should widen financial support and increase funding levels. Third, more attention should be directed to further improving the design of URRBMI, which could include providing greater government subsidies and increasing its share of the premiums, to gradually eliminate the existing two-level premium structure. Fourth, more targeted policies for the poor are needed, including reducing out-of-pocket medical costs and facilitating and expanding family physician contract programs to improve their access to healthcare. Fifth, increasing the coverage of outpatient service and providing chronic disease-related preventive services packages at the primary health care facilities could reduce the overuse among the rich and underuse among the poor, so as to improve equitable access to inpatient care. In addition, it should be clearly noted that the integration reform alone is unlikely to eliminate inequity in inpatient service utilization, coordinated inter-government strategies aimed to reduce socioeconomic inequity in income and social welfare are also needed.

## Additional files

Additional file 1: Description of the survey population. (DOCX 22 kb)

Additional file 2: The actual reimbursement rate for inpatient service of different medical insurance scheme (%). (DOCX 16 kb)

## Data Availability

The data that support the findings of this study are available from Centre of Health Statistics and Information, National Health Commission of the People’s Republic of China, through its confidential data center, which were used under license for the current study, and thus are not available to the public. Requests for access to the data should be directed to Centre of Health Statistics and Information, National Health Commission of the People’s Republic of China.

## Abbreviations

CI: Concentration index
HI: Horizontal inequity index
NRCMS: The New Rural Cooperative Medical Scheme
SBMI: Social Basic Medical Insurance
UEBMI: Urban Employee Basic Medical Insurance
UHC: Universal health coverage
URBMI: Urban Resident Basic Medical Insurance
URRBMI: Urban-Rural Residents Basic Medical Insurance
USBMI: Uniform Social Basic Medical Insurance
