# Supplementary material for: Can integration reduce inequity in healthcare utilization? Evidence and hurdles in China
Source: BMC Health Serv Res. 2019 Sep 10;19:654. doi: 10.1186/s12913-019-4480-8 (PMC6734466; doi:10.1186/s12913-019-4480-8)
Supplement: Supplementary file 1 — Description of the survey population. (DOCX 22 kb) [file 12913_2019_4480_MOESM1_ESM.docx]

**Additional file 1: Table S1** Description of the survey population

|  | Pilot integrated area  (N=49365) | |  | Non-integrated area  (N=77165) | | |
| --- | --- | --- | --- | --- | --- | --- |
|  | N | % |  | N | | % |
| **Sex and Age** |  |  |  |  | |  |
| **Male** |  |  |  |  | |  |
| 15-24 | 2759 | 5.59% |  | 4001 | | 5.18% |
| 25-34 | 3458 | 7.00% |  | 4675 | | 6.06% |
| 35-44 | 4119 | 8.34% |  | 6510 | | 8.44% |
| 45-54 | 4508 | 9.13% |  | 7666 | | 9.93% |
| 55-64 | 4978 | 10.08% |  | 7684 | | 9.96% |
| 65- | 4334 | 8.78% |  | 7020 | | 9.10% |
| **Female** |  |  |  |  | |  |
| 15-24 | 2688 | 5.45% |  | 3882 | | 5.03% |
| 25-34 | 3737 | 7.57% |  | 5239 | | 6.79% |
| 35-44 | 4326 | 8.76% |  | 6979 | | 9.04% |
| 45-54 | 4944 | 10.02% |  | 8267 | | 10.71% |
| 55-64 | 5013 | 10.15% |  | 8197 | | 10.62% |
| 65- | 4501 | 9.12% |  | 7045 | | 9.13% |
| **Chronic disease** |  |  |  |  | |  |
| Yes | 12035 | 24.38% |  | 18171 | | 23.55% |
| No | 37330 | 75.62% |  | 58994 | | 76.45% |
| **Limitation of daily activities** |  |  |  |  | |  |
| Yes | 2839 | 5.75% |  | 4737 | | 6.14% |
| No | 46526 | 94.25% |  | 72428 | | 93.86% |
| **Self-assessment health** |  |  |  |  | |  |
| Very poor | 2420 | 4.90% |  | 4394 | | 5.69% |
| Poor | 3285 | 6.65% |  | 6082 | | 7.88% |
| Medium | 6214 | 12.59% |  | 10512 | | 13.62% |
| Good | 14835 | 30.05% |  | 22825 | | 29.58% |
| Very good | 22611 | 45.80% |  | 33352 | | 43.22% |
| **Education** |  |  |  |  | |  |
| Illiterate | 5762 | 11.67% |  | 8793 | | 11.40% |
| Primary school | 27328 | 55.36% |  | 45339 | | 58.76% |
| Secondary school | 9535 | 19.32% |  | 14518 | | 18.81% |
| University and above | 6740 | 13.65% |  | 8515 | | 11.03% |
| **Occupation status** |  |  |  |  | |  |
| Unemployment | 8207 | 16.63% |  | 10820 | | 14.02% |
| Student | 2357 | 4.77% |  | 3236 | | 4.19% |
| Peasant | 12506 | 25.33% |  | 30499 | | 39.52% |
| Worker | 4839 | 9.80% |  | 4509 | | 5.84% |
| Business | 6384 | 12.93% |  | 7757 | | 10.05% |
| Manager | 10299 | 20.86% |  | 13484 | | 17.47% |
| Other | 4773 | 9.67% |  | 6860 | | 8.89% |
| **Marital status** |  |  |  |  | |  |
| Other | 10866 | 22.01% |  | 15451 | | 20.02% |
| Married | 38499 | 77.99% |  | 61714 | | 79.98% |
| **Household income** |  |  |  |  | |  |
| Quintile Ⅰ(Poorest) | 9887 | 20.03% |  | 15437 | | 20.01% |
| Quintile Ⅱ | 9860 | 19.97% |  | 15442 | | 20.01% |
| Quintile Ⅲ | 9886 | 20.03% |  | 15421 | | 19.98% |
| Quintile Ⅳ | 9895 | 20.04% |  | 15433 | | 20.00% |
| Quintile Ⅴ (Richest) | 9837 | 19.93% |  | 15432 | | 20.00% |
| **Medical insurance** |  |  |  |  | |  |
| UEBMI | 13564 | 27.48% |  | 18294 | | 23.71% |
| URBMI | ---- | ---- |  | 8046 | | 10.43% |
| NRCMS | ---- | ---- |  | 43700 | | 56.63% |
| USBMI | 2700 | 5.47% |  | ---- | | ---- |
| URRBMI | 28447 | 57.63% |  | ---- | | ---- |
| Mixed-insurance | 2893 | 5.86% |  | 4541 | | 5.88% |
| Uninsured | 1761 | 3.57% |  | 2584 | | 3.35% |
| **Distance to the nearest health facilities** |  |  |  |  | |  |
| <1km | 29328 | 59.41% |  | 48979 | | 63.47% |
| 1-4km | 18779 | 38.04% |  | 26259 | | 34.03% |
| ≥5km | 1258 | 2.55% |  | 1927 | | 2.50% |
| **Time to the nearest health facilities** |  |  |  |  | |  |
| <15 min | 37860 | 76.69% |  | 57336 | | 74.30% |
| 15-29min | 8885 | 18.00% |  | 13978 | | 18.11% |
| ≥30 min | 2620 | 5.31% |  | 5851 | | 7.58% |
| **Preferred health facilities** | | |  |  | |  |
| Primary | 38237 | 77.46% |  | 62733 | | 81.30% |
| Non-primary | 11128 | 22.54% |  | 14432 | | 18.70% |
| **Residence** |  |  |  |  | |  |
| Urban | 26648 | 53.98% |  | 37707 | | 48.87% |
| Rural | 22717 | 46.02% |  | 39458 | | 51.13% |
| **Region** |  |  |  |  | |  |
| Eastern | 27828 | 56.37% |  | | 23911 | 30.99% |
| Middle | 5996 | 12.15% |  | 28885 | | 37.43% |
| Western | 15541 | 31.48% |  | 24369 | | 31.58% |

Note: QuintileⅠwas the poorest 20%,and the Quintile Ⅴ was the richest 20%
