# Supplementary material for: Can integration reduce inequity in healthcare utilization? Evidence and hurdles in China
Source: BMC Health Serv Res. 2019 Sep 10;19:654. doi: 10.1186/s12913-019-4480-8 (PMC6734466; doi:10.1186/s12913-019-4480-8)
Supplement: Supplementary file 2 — The actual reimbursement rate for inpatient service of different medical insurance scheme (%). (DOCX 16 kb) [file 12913_2019_4480_MOESM2_ESM.docx]

**Additional file 2: Table S2 The actual reimbursement rate for inpatient service of different medical insurance scheme (%)**

|  | **Quintile Ⅰ** | **Quintile Ⅱ** | **Quintile Ⅲ** | **Quintile Ⅳ** | **Quintile Ⅴ** | **Total** |
| --- | --- | --- | --- | --- | --- | --- |
| **UEBMI** | 66.1 | 67.0 | 67.0 | 67.3 | 65.9 | 66.8 |
| **URBMI** | 53.5 | 49.1 | 48.9 | 45.7 | 44.3 | 47.3 |
| **NRCMS** | 59.4 | 52.8 | 52.0 | 48.1 | 45.7 | 52.0 |
| **USBMI** | 62.2 | 57.8 | 64.0 | 59.0 | 59.7 | 60.1 |
| **URRBMI** | 57.6 | 51.0 | 48.2 | 46.8 | 44.6 | 49.8 |
| **Mixed-insurance** | 66.7 | 54.4 | 57.1 | 53.5 | 57.3 | 56.8 |

Note: QuintileⅠwas the poorest 20%,and the Quintile Ⅴ was the richest 20%
